# The impact of glucose on mitochondria and life span is determined by the integrity of proline catabolism in *Caenorhabditis elegans*

**DOI:** 10.1016/j.jbc.2023.102881

**Published:** 2023-01-07

**Authors:** Xi Feng, Xinyu Wang, Lei Zhou, Shanshan Pang, Haiqing Tang

**Affiliations:** 1 School of Life Sciences, Chongqing University, Chongqing, China; 2 State Key Laboratory of Silkworm Genome Biology, Key Laboratory of Sericultural Biology and Genetic Breeding, Ministry of Agriculture and Rural Affairs, College of Sericulture, Textile and Biomass Sciences, Southwest University, Chongqing, China

**Keywords:** proline catabolism, glucose metabolism, mitochondria, ROS, life span, pentose phosphate pathway, *C. elegans*, G6P, glucose-6-phosphate, G6PDH, G6P dehydrogenase, HG, high-glucose, IDH, isocitrate dehydrogenase, NGM, nematode-growth medium, P5C, pyrroline-5-carboxylate, P5CDH, pyrroline-5-carboxylate dehydrogenase, PPP, pentose phosphate pathway, PRODH, proline dehydrogenase, PYK, pyruvate kinase, ROS, reactive oxygen species

## Abstract

Mutations in genes involved in mitochondrial proline catabolism lead to the rare genetic disorder hyperprolinemia in humans. We have previously reported that mutations of proline catabolic genes in *Caenorhabditis elegans* impair mitochondrial homeostasis and shorten life span, and that these effects surprisingly occur in a diet type–dependent manner. Therefore, we speculated that a specific dietary component may mitigate the adverse effects of defective proline catabolism. Here, we discovered that high dietary glucose, which is generally detrimental to health, actually improves mitochondrial homeostasis and life span in *C. elegans* with faulty proline catabolism. Mechanistically, defective proline catabolism results in a shift of glucose catabolism toward the pentose phosphate pathway, which is crucial for cellular redox balance. This shift helps to maintain mitochondrial reactive oxygen species homeostasis and to extend life span, as suppression of the pentose phosphate pathway enzyme GSPD-1 prevents the favorable effects of high glucose. In addition, we demonstrate that this crosstalk between proline and glucose catabolism is mediated by the transcription factor DAF-16. Altogether, these findings suggest that a glucose-rich diet may be advantageous in certain situations and might represent a potentially viable treatment strategy for disorders involving impaired proline catabolism.

Amino acids serve as the building blocks of proteins and also serve as an energy resource through their catabolism (1). Mitochondria are the primary organelles for the catabolism of many amino acids. Defective amino acid catabolism leads to abnormal accumulation of certain metabolic intermediates in mitochondria, which impairs mitochondrial homeostasis, cellular functions, and ultimately organismal health (2, 3, 4). Mutations in genes involved in mitochondrial amino acid catabolism lead to a group of human genetic diseases belonging to amino acid metabolic disorders (5, 6). Although the mitochondrial and cellular damages caused by faulty amino acid catabolism have been recognized, how cells adapt to or alleviate these stresses remains largely unknown.

Proline catabolism consists of two sequential enzymatic reactions catalyzed by proline dehydrogenase (PRODH) and pyrroline-5-carboxylate (P5C) dehydrogenase (P5CDH), respectively. Mutations in *p5cdh* lead to the rare genetic disease hyperprolinemia type II, which is characterized by mitochondrial accumulation of the intermediate P5C and is associated with seizures and varying degrees of mental retardation in patients (7, 8). Our prior research in *Caenorhabditis elegans* demonstrated that mitochondria are the primary target of P5C accumulation, as the *p5cdh* mutation increases mitochondrial reactive oxygen species (ROS), impairs mitochondrial homeostasis, and thereby shortens life span (2, 3). Notably, these effects are conserved, as P5CDH inhibition or PRODH overexpression (both of which increase P5C levels) induces mitochondrial ROS overproduction and cell death in cultured tumor cells (9, 10, 11). Thus, understanding how cells adapt to P5C buildup may have important implications for treating various disorders associated with defective proline metabolism.

Diet has a profound effect on animal physiology. We reported that defective proline catabolism impairs mitochondrial function and life span in *C. elegans* in a diet type–dependent manner. It only occurs when *C. elegans* are fed *Escherichia coli* B strains (such as OP50) but not K-12 strains (such as HT115) (2). This phenomenon is known as “gene–diet pair,” which indicates that the effects of a genetic mutation can only be observed on specific diets (12). Identifying specific dietary components that interact with the gene and determining how they interact are crucial for understanding the gene–diet pair. In this study, we postulated that a specific dietary component of K-12 bacteria may protect animals from the deleterious effects of defective proline catabolism. Surprisingly, we found that the high-glucose (HG) diet, which is normally detrimental to mitochondrial homeostasis and life span, completely restores mitochondrial homeostasis and life span in *C. elegans* with faulty proline catabolism. This effect is dependent on DAF-16-mediated induction of the pentose phosphate pathway (PPP) enzyme, which ameliorates the ROS overload caused by P5C buildup. Our findings suggest that HG may be advantageous in certain genetic contexts and that a glucose-rich diet is a viable and simple strategy for coping with human disorders caused by defective proline catabolism.

## Results and discussion

### Dietary glucose protects *C. elegans* from defective proline catabolism

**Figure 1:**
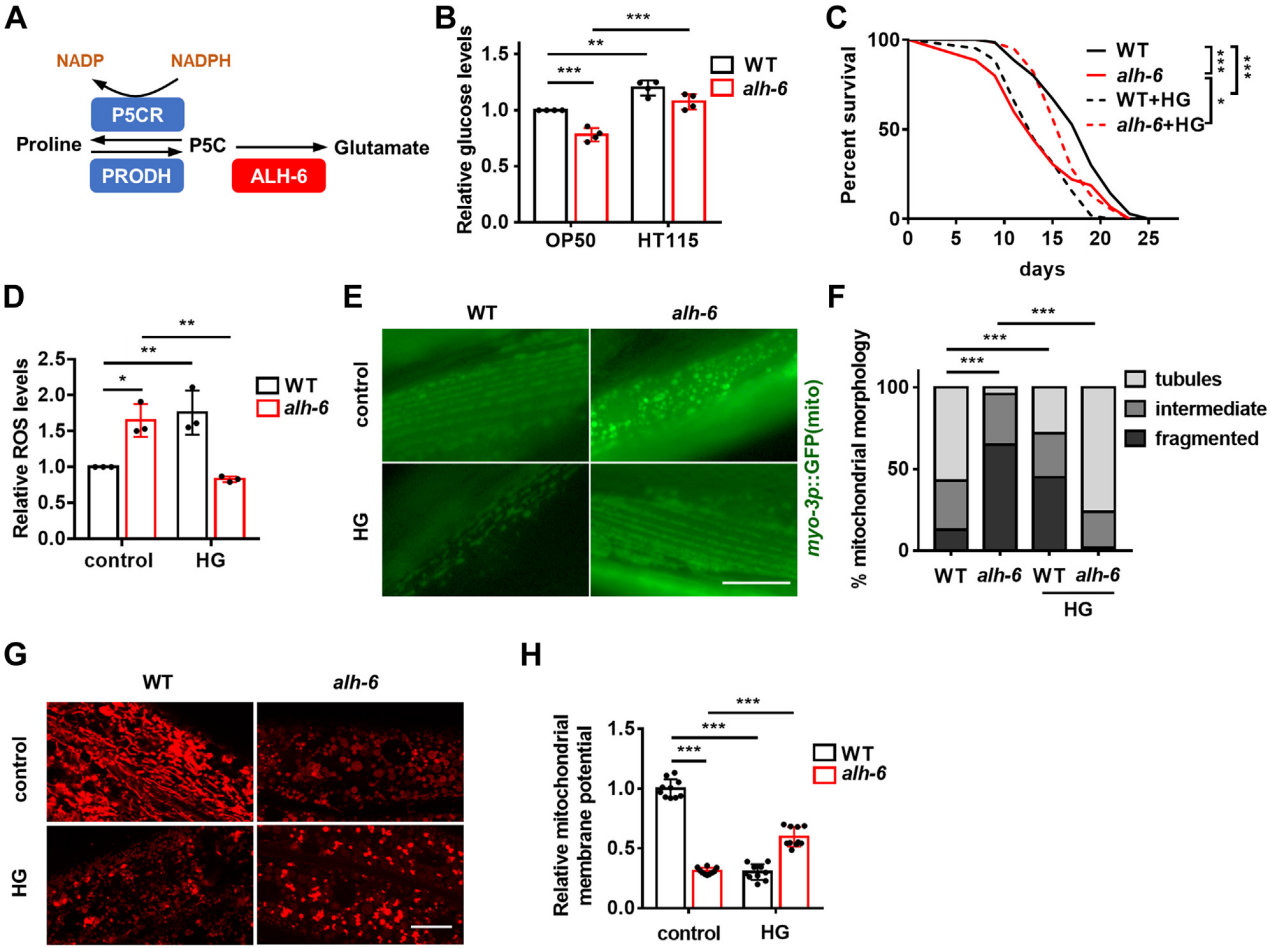
**The HG diet mitigates mitochondrial abnormalities and restores life span in *alh-6* mutants.***A*, schematic of proline metabolism in *Caenorhabditis elegans*. *B*, glucose levels in WT and *alh-6* mutants fed OP50 and HT115 bacterial diets. Data are from four independent experiments and normalized to the control values within the same experiment. *C*–*F*, the effects of the HG diet on life span (*C*), ROS levels (*D*), mitochondrial morphology, as indicated by *myo-3p*::GFP (mito) (*E* and *F*), and mitochondrial membrane potential (*G* and *H*) in WT and *alh-6* mutants fed OP50 bacteria. *D*, data are from three independent experiments and normalized to the control values within the same experiment. *E* and *G*, representative images. The scale bar represents 12 μm. *F* and *H*, semiquantification data. Approximately n = 200 mitochondria per condition in (*F*) and n = 10 worms per condition in (*H*). Data are represented as mean ± SD. ∗*p* < 0.05, ∗∗*p* < 0.01, and ∗∗∗*p* < 0.001. HG, high-glucose; ROS, reactive oxygen species.

In *C. elegans*, mitochondrial PRODH and P5CDH are encoded by the genes *prodh* and *alh-6*, respectively (Fig. 1*A*). We have previously reported that the *alh-6* mutation disrupts mitochondrial homeostasis, impairs stress response, and accelerates aging (2, 3, 13). These phenotypes are only observed when *C. elegans* are fed OP50 bacteria but not HT115 bacteria, likely because of the P5C overload resulting from increased *prodh* expression in OP50-fed worms (2). Although *prodh* expression is relatively lower in worms fed HT115, we reasoned that the *alh-6* mutation could also result in P5C accumulation in these phenotypically normal mutants and that certain components of HT115 bacteria may protect the host from such metabolic stress.

In terms of nutritional composition, the carbohydrate contents of OP50 and HT115 bacteria differ the most. Significantly, more carbohydrates are found in HT115 bacteria than in OP50 bacteria (14). In addition, we found that *C. elegans*-fed HT115 bacteria have higher levels of glucose, the primary carbohydrate, than those fed OP50 bacteria (Fig. 1*B*). Surprisingly, *alh-6* mutants contained much less glucose than WT controls, especially when fed the OP50 diet (Fig. 1*B*), which correlates with their life span. These data imply that *alh-6* mutants may live longer on a high carbohydrate/HG diet. As such, we fed them an HG diet, a treatment that normally shortens the life span of WT *C. elegans* (15). Indeed, the HG diet restored glucose levels in *alh-6* mutants fed the OP50 diet (Fig. S1*A*). Notably, while the HG diet reduced the life span of WT worms as previously reported (15), it fully restored the life span (Fig. 1*C* and Table S1) and stress resistance (Fig. S1*B* and Table S1) of *alh-6* mutants, suggesting that HG becomes beneficial to the life span when proline catabolism is defective.

As the *alh-6* mutation shortens life span through excessive mitochondrial ROS production that impairs mitochondrial homeostasis (2), we examined whether the HG diet protects the mitochondria against defective proline catabolism. Intriguingly, the effects of the HG diet on the mitochondria of WT and *alh-6* mutants were opposite. It caused ROS overproduction (Fig. 1*D*), mitochondrial fragmentation (Fig. 1, *E* and *F*), and reduction of mitochondrial membrane potential (Fig. 1, *G* and *H*) in WT worms, but it normalized ROS production and mitochondrial phenotypes in *alh-6* mutants fed an OP50 diet (Fig. 1, *D*–*H*), which correlates with life span. Thus, HG improves mitochondrial homeostasis in response to faulty proline catabolism, which likely contributes to its effect on life span extension.

### Defective proline catabolism alters glucose catabolism

Why does the HG diet affect WT and *alh-6* mutants oppositely? Glucose catabolism plays dual roles in ROS homeostasis. On the one hand, HG is associated with ROS overproduction *via* several mechanisms. For example, glycolysis and the Krebs cycle supply NADH for oxidative phosphorylation, which generates ROS as a byproduct. Also, HG can activate NADPH oxidase that contributes to ROS production (16). On the other side, glucose catabolism can balance ROS levels *via* the alternative PPP, the primary source of the potent cellular reducing power NADPH (17, 18). Therefore, we postulated that faulty proline catabolism may alter glucose catabolism, resulting in divergent responses to the HG diet in WT and *alh-6* mutant worms.

**Figure 2:**
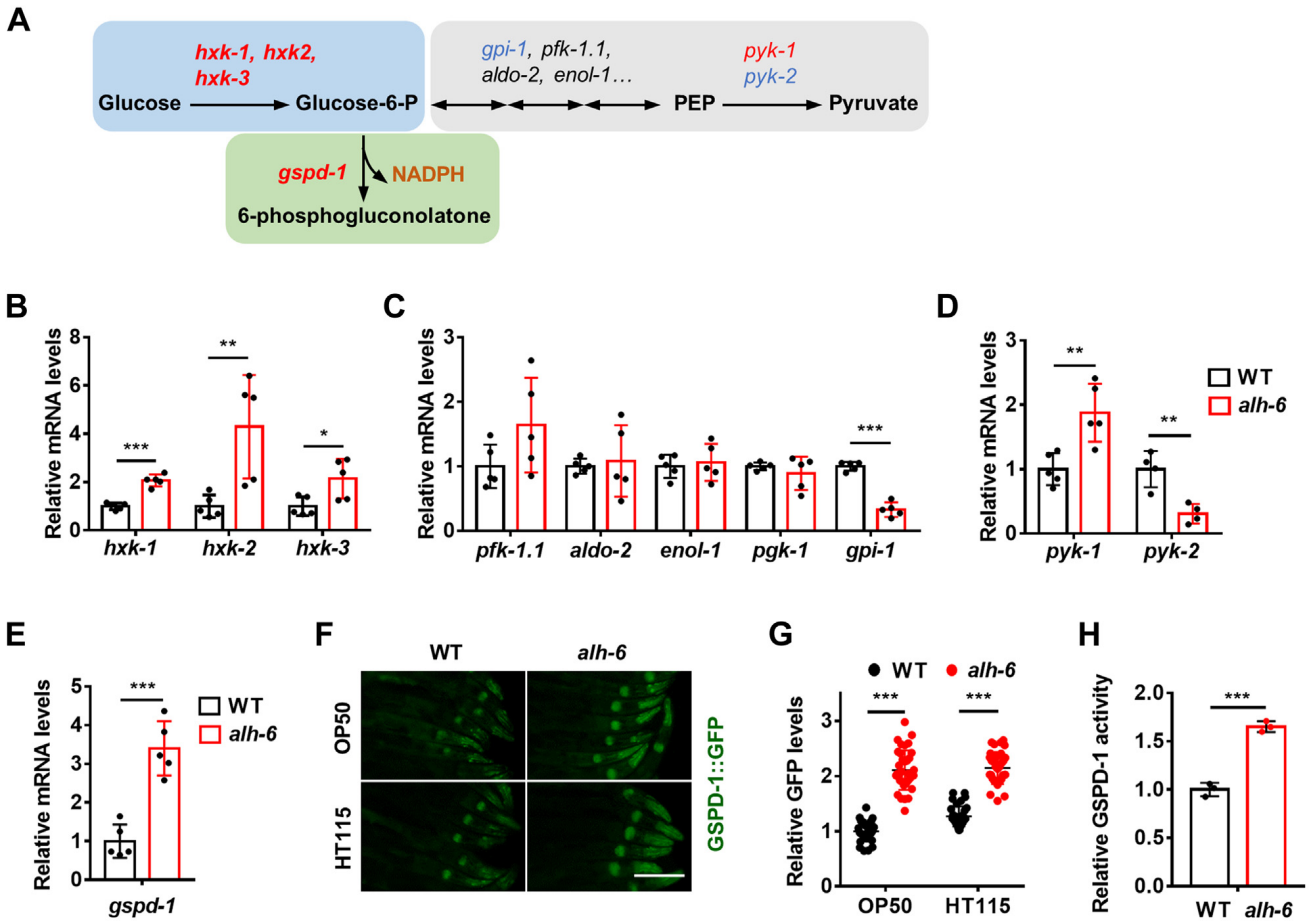
**The *alh-6* mutation alters glucose catabolism.***A*, schematic of glucose catabolism in *Caenorhabditis elegans*. Genes in *red* indicate upregulation, whereas genes in *blue* indicate downregulation in *alh-6* mutants fed OP50 diet. *B*–*E*, the mRNA expression of genes that are common for glycolysis and the PPP (*B*), bidirectional for glycolysis and gluconeogenesis (*C*), specific for glycolysis (*D*), and specific for the PPP (*E*) in *alh-6* mutants fed OP50 bacteria. n = 5 biologically independent samples per condition. *F* and *G*, the effects of the *alh-6* mutation on GSPD-1::GFP expression in worms fed OP50 and HT115 diets. *F*, representative images. The scale bar represents 100 μm. *G*, quantification data. n = 31 worms per condition. *H*, the effects of the *alh-6* mutation on GSPD-1 activity in worms fed OP50. n = 3 biologically independent samples per condition. Data are represented as mean ± SD. ∗*p* < 0.05, ∗∗*p* < 0.01, and ∗∗∗*p* < 0.001. PPP, pentose phosphate pathway.

As such, we next analyzed the expression of genes involved in glucose catabolism using quantitative PCR (Fig. 2*A*). Hexokinase, the rate-limiting enzyme of glucose catabolism, converts glucose to glucose-6-phosphate (G6P), which is then metabolized through glycolysis or the PPP (Fig. 2*A*). All three hexokinase genes in *C. elegans* (*hxk-1*, *hxk-2*, and *hxk-3*) were upregulated by the *alh-6* mutation (Fig. 2, *A* and *B*), indicating an increase in glucose catabolism as a result of defective proline catabolism. Next, we investigated the glycolytic genes, the majority of which were not significantly affected by the *alh-6* mutation (Fig. 2, *A* and *C*). Since most glycolytic enzymes in *C. elegans* are bidirectional for both glycolysis and gluconeogenesis (Fig. 2*A*), it is difficult to establish a correlation between their expression and glucose flux. Pyruvate kinases (PYK-1 and PYK-2) catalyze the unidirectional step of glycolysis, and in *alh-6* mutants, *pyk-1* was modestly upregulated, whereas *pyk-2* was significantly downregulated (Fig. 2, *A* and *D*). These data suggest that glycolysis is unlikely to be induced by the *alh-6* mutation.

G6P dehydrogenase (G6PDH), encoded by *gspd-1* in *C. elegans*, is the first and rate-limiting enzyme of the PPP (Fig. 2*A*). We observed a substantial upregulation of *gspd-1* in *alh-6* mutants (Fig. 2, *A* and *E*). To confirm this, we constructed a translational GFP reporter for *gspd-1.* The GSPD-1::GFP signals were most abundant in the pharyngeal muscle, head muscle, and seam cells, and substantially less abundant in the intestine (Fig. S2, *A* and *C*). Consistent with the quantitative PCR data, GSPD-1::GFP was also induced in *alh-6* mutants (Fig. S2, *F* and *G*). The enzyme activity of GSPD-1 was also examined, which correlated with its expression (Fig. 2*H*). Together, these data suggest that the concurrent induction of hexokinase and GSPD-1 may direct the glucose flux into the PPP when the proline catabolism is compromised.

As the HG diet could increase ROS levels (Fig. 1*D*), we wondered if it also engages the PPP. However, the HG diet only had a marginal effect on the expression of GSPD-1::GFP, which was much less pronounced than that of the *alh-6* mutation (Fig. S2, *D* and *E*), suggesting that the PPP activation is not a general response to an increase in ROS.

We also examined the expression of glucose metabolic genes in *alh-6* mutants fed HT115 bacteria, when these mutants displayed normal mitochondria and life span. Intriguingly, while the hexokinase genes only showed little or no increases (Fig. S2*F*), the expression of *gspd-1* was still significantly elevated in *alh-6* mutants (Fig. S2*F*), which was also confirmed by GSPD-1::GFP (Fig. 2, *F* and *G*), indicating that *gsdp-1* induction by *alh-6* mutation is diet independent. Collectively, these findings imply that defective proline catabolism alters glucose catabolism that may direct glucose toward the PPP.

### GSPD-1 protects *C. elegans* from defective proline catabolism

**Figure 3:**
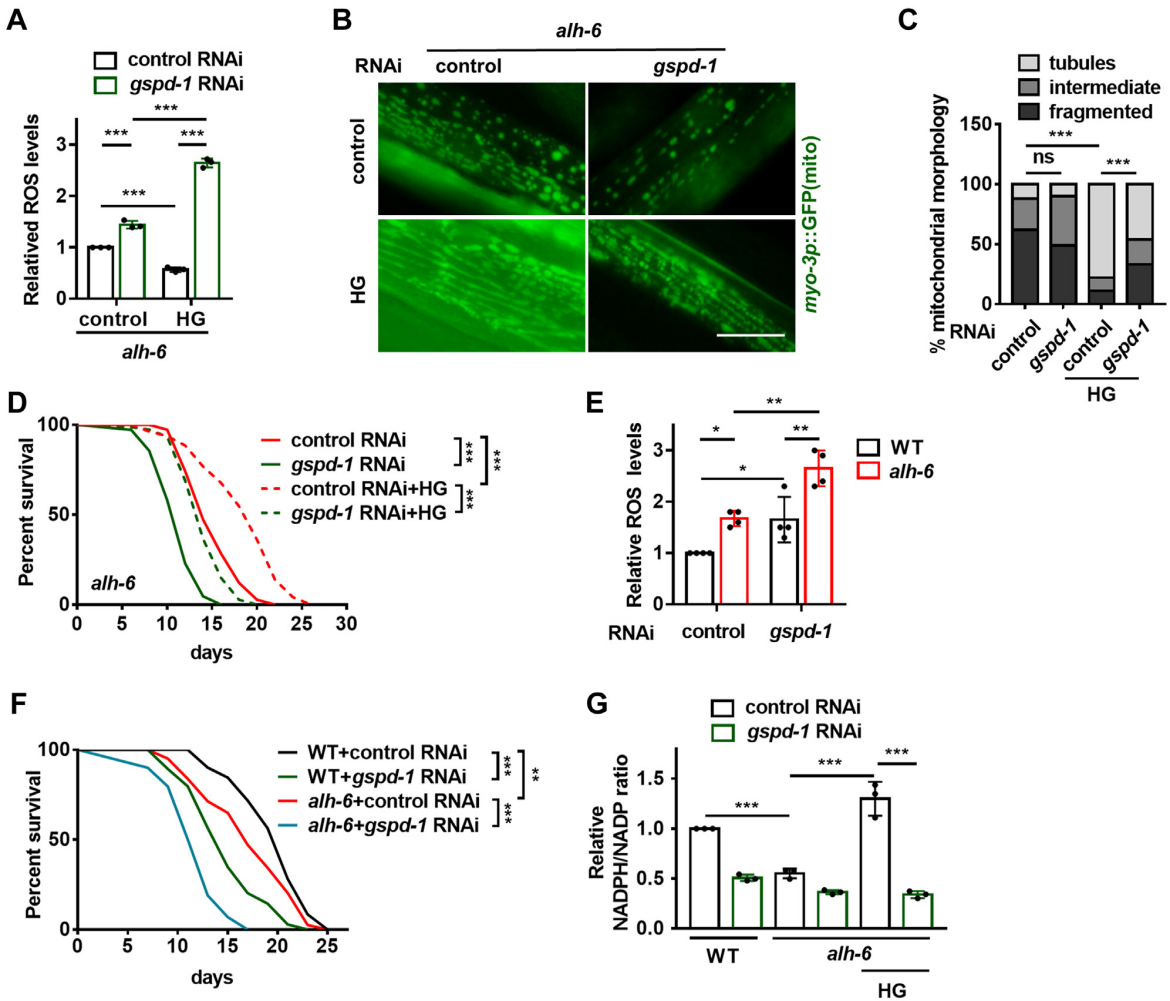
**GSPD-1 is required for the beneficial effects of the HG diet.***A*–*D*, *gspd-1* RNAi suppresses the protective effects of the HG diet on ROS production (*A*), mitochondrial morphology (*B* and *C*), and life span (*D*) in *alh-6* mutants fed OP50(xu363) bacteria. *A*, data are from three independent experiments and normalized to the control values within the same experiment. *B*, shows the representative images for mitochondrial morphology (the scale bar represents 12 μm). *C*, shows the semiquantification data. Approximately n = 200 mitochondria per condition. *E* and *F*, in the absence of HG, *gspd-1* RNAi worsens the ROS overproduction (*E*) and life span (*F*) of *alh-6* mutants fed OP50(xu363) bacteria. *E*, data are from four independent experiments and normalized to the control values within the same experiment. *G*, the effects of *gspd-1* RNAi on the NADPH/NADP ratio in *alh-6* mutants fed the HG diet. Data are from three independent experiments and normalized to the control values within the same experiment. Data are represented as mean ± SD. ∗*p* < 0.05, ∗∗*p* < 0.01, and ∗∗∗*p* < 0.001. HG, high-glucose; ROS, reactive oxygen species.

The reaction catalyzed by GSPD-1 produces NADPH, a critical redox balancer of the cell. As increased ROS levels are the cause of mitochondrial fragmentation and shortened life span in *alh-6* mutants (2), we postulated that the shift to the PPP is an adaptive response aimed at restoring ROS homeostasis in these mutants. When cellular glucose is abundant, as in the context of the HT115 or HG diet, glucose flux into the PPP is protective. If this is true, then *gspd-1* suppression would abolish the beneficial effects of HG in *alh-6* mutants. The RNAi-feeding bacteria in *C. elegans* is usually the HT115 strain, on which the *alh-6* mutants aged normally; therefore, we utilized the modified RNAi-compatible OP50 strain OP50(xu363) as an alternative RNAi delivery strain (19). We verified that *alh-6* mutants fed OP50(xu363) had a shorter life span, which was rescued by the HG feeding (Fig. S3*A* and Table S1), and that *gspd-1* RNAi *via* OP50(xu363) was effective (Fig. S3*B*). Furthermore, we found that *gspd-1* knockdown abolished the protective effects of the HG diet on ROS production (Fig. 3*A*), mitochondrial morphology (Fig. 3, *B* and *C*), membrane potential (Fig. S3, *C* and *D*), and life span (Fig. 3*D* and Table S1) in *alh-6* mutants, suggesting that the HG diet requires the PPP enzyme to protect against faulty proline catabolism. In addition, this effect is specific to the oxidative arm of the PPP, which is mediated by GSPD-1/G6PDH, as RNAi inactivation of key enzymes in the nonoxidative arm of the PPP (Fig. S3*E*) had very minor effects on the life span of the *alh-6* mutants fed the HG diet (Fig. S3, *F* and *G* and Table S1).

Other metabolic enzymes, including folate metabolic enzyme (20), malic enzymes (21, 22), and isocitrate dehydrogenase (IDH) (23), may also contribute to NADPH production and thereby protect against ROS. As such, we used RNAi to knock down the relevant metabolic enzymes (Fig. S3*H*) and found that the majority of these enzymes had no effects on ROS levels (Fig. S3*I*). Only *idh-1/Idh1* RNAi increased ROS levels modestly in *alh-6* mutants fed the HG diet (Fig. S3*I*), but its effect was much less pronounced than that of *gspd-1* RNAi (Fig. 3*A*). Thus, these data suggest that HG primarily utilizes the PPP to combat ROS in the context of faulty proline catabolism.

As *gspd-1* was induced by *alh-6* mutation in the absence of HG diet, we speculated that *gspd-1* suppression would exacerbate the detrimental phenotypes of *alh-6* mutants. Indeed, *gspd-1* RNAi worsened the ROS, mitochondrial membrane potential, and life span abnormalities of *alh-6* mutants (Figs. 3, *E* and *F*, S3, *C* and *D* and Table S1), further supporting that *gspd-1* induction is protective when *alh-6* is mutated. The mitochondrial fragmentation of *alh-6* mutants was not further impaired by *gspd-1* RNAi (Fig. 3, *B* and *C*), implying that this phenotype may be too severe to be altered further.

Next, as GSPD-1 mediates the production of NADPH, we examined the ratio of NADPH/NADP. *alh-6* mutants exhibited a decreased ratio of NADPH/NADP, which was restored by HG (Fig. 3*G*). More notably, the beneficial effect of the HG diet on NADPH/NADP was entirely eliminated by *gspd-1* RNAi (Fig. 3*G*), indicatin/g that HG produces NADPH *via* GSPD-1 in the presence of faulty proline catabolism. NADPH can alleviate cellular ROS *via* multiple mechanisms, the most prominent of which is glutathione recycling (GSH/GSSG). However, HG had no effect on the ratio of GSH/GSSG in both WT and *alh-6* mutants (Fig. S3*J*), showing that NADPH may not influence ROS homeostasis *via* glutathione recycling under HG conditions. While further investigation is required to determine how HG-induced NADPH combats ROS, these data support a model in which HG flux *via* GSPD-1 produces NADPH that is crucial for alleviating ROS burden in animals with faulty proline catabolism.

### DAF-16 mediates the communication between proline catabolism and the PPP

**Figure 4:**
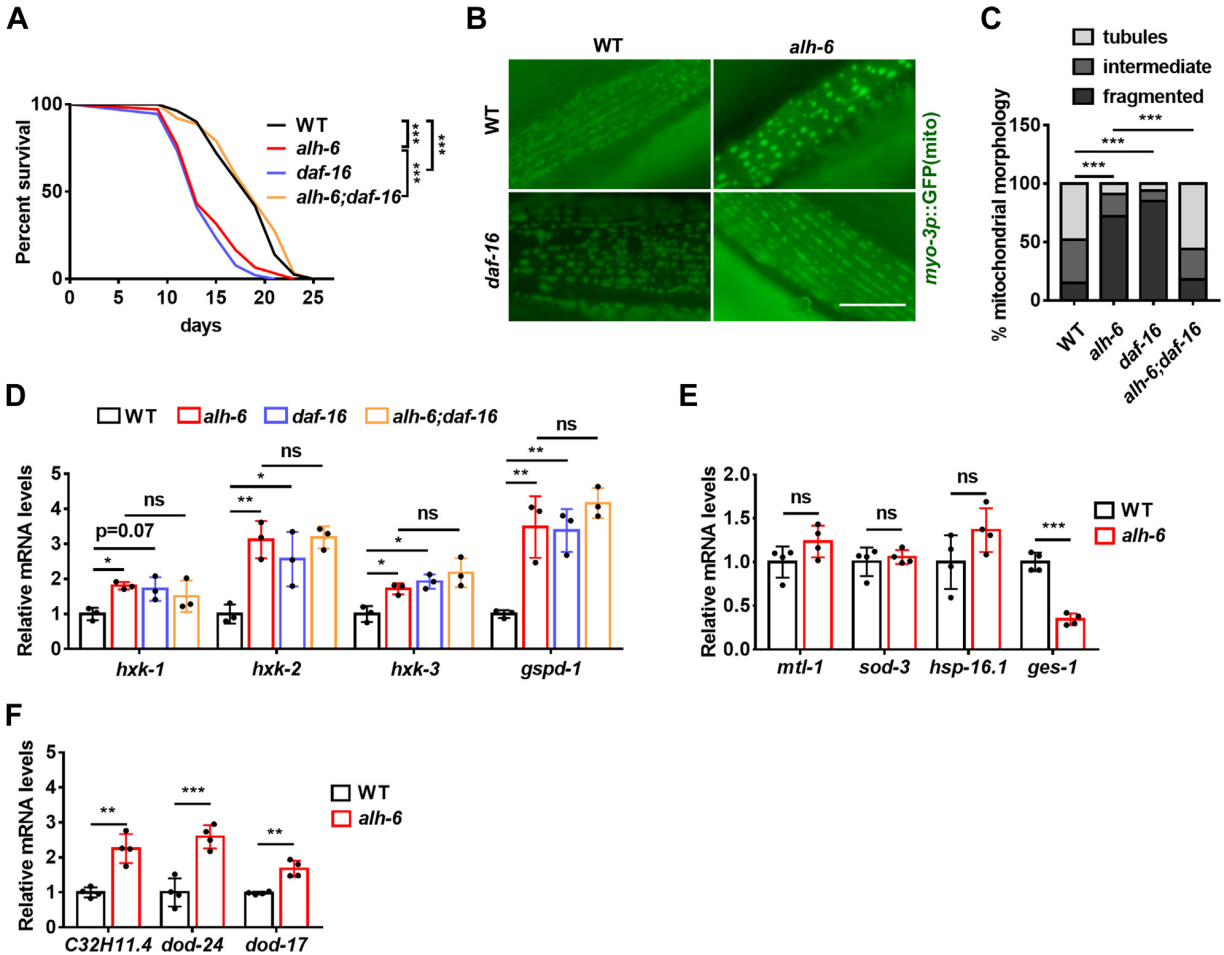
**DAF-16 mediates the communication between proline and glucose catabolism.***A*–*C*, the *daf-16* mutation restores the life span (*A*) and mitochondrial morphology (*B* and *C*) of *alh-6* mutants fed OP50 bacteria. *B*, representative images of mitochondrial morphology. The scale bar represents 12 μm. *C*, semiquantification data. Approximately n = 200 mitochondria per condition. *D*, the effects of the *daf-16* mutation on *hxk* and *gspd-1* expression. n = 3 biologically independent samples per condition. *E* and *F*, the effects of the *alh-6* mutation on the expression of DAF-16 class I (*E*) and class II targets (*F*). n = 4 biologically independent samples per condition. Data are represented as mean ± SD. ∗*p* < 0.05, ∗∗*p* < 0.01, and ∗∗∗*p* < 0.001.

Next, we explored how defective proline catabolism upregulates the PPP enzyme. We inquired as to the involvement of any well-known transcription factors that govern life span, such as SKN-1 (24), HSF-1 (25, 26), and DAF-16 (27, 28). SKN-1, the transcription factor that regulates detoxification response and life span, is activated in the muscle of *alh-6* mutants (2). However, SKN-1 is not involved in *alh-6*-mediated life span regulation by the *alh-6* mutation (2). We also found that the *alh-6* mutation still shortened the life span of *hsf-1* mutants (Fig. S4*A* and Table S1). DAF-16/FOXO is the most well-known and conserved transcription factor for longevity (29). While it was commonly recognized that the *daf-16* mutation shortens the life span of *C. elegans*, it surprisingly restored the life span of *alh-6* mutants fed OP50 bacteria (Fig. 4*A* and Table S1). Further examination of the mitochondrial phenotype revealed that the *daf-16* mutation caused mitochondrial fragmentation in WT worms but improved mitochondrial morphology in *alh-6* mutants fed OP50 bacteria (Fig. 4, *B* and *C*), which correlates with the life span data.

How does the *daf-16* mutation rescue the mitochondrial phenotype and life span of *alh-6* mutants? We tested the possibility that the *daf-16* mutation is responsible for the induction of glucose catabolic genes. Similar to *alh-6* mutants, *daf-16* mutants exhibited enhanced expression of *hxk* genes and *gspd-1* (Fig. 4*D*), indicating that DAF-16 normally suppresses these genes. More importantly, these effects were not additive to those of the *alh-6* mutation (Fig. 4*D*), suggesting that the *alh-6* and *daf-16* mutations act in the same pathway to regulate these glucose catabolic genes. Intriguingly, it was observed that DAF-16 stimulates gluconeogenesis genes but not glucose catabolic genes like *hxk* in starved *C. elegans* larvae (30). This implies that DAF-16 may regulate glucose metabolism differentially to cope with specific metabolic challenges under varied nutritional or developmental conditions.

Next, we investigated whether the *alh-6* mutation regulates DAF-16 activity. The *alh-6* mutation did not affect the nuclear occupancy of DAF-16::GFP (Fig. S4, *B*–*E*), suggesting the subcellular location of DAF-16 is not regulated. We next examined the expression of DAF-16 target genes, which are divided into class I (DAF-16-upregulated) and class II (DAF-16-downregulated) (31, 32). The expression of many DAF-16 class I targets was seemingly not affected by the *alh-6* mutation (Fig. 4*E*), which was confirmed by the well-established DAF-16 class I reporter s*od-3p*::GFP (Fig. S4, *F* and *G*). Intriguingly, the *alh-6* mutation increased the expression of several DAF-16 class II target genes (DAF-16-downregulated) (Fig. 4*F*). Since *hxk* genes and *gspd-1* are likewise suppressed by DAF-16, these glucose catabolic genes also belong to DAF-16 class II targets. Therefore, the *alh-6* mutation may preferentially promote DAF-16 class II target genes, including *hxk* and *gspd-1*. As DAF-16 was found to inhibit its class II targets indirectly (32), other factor(s) may act downstream of DAF-16 to mediate the activation of glucose catabolic genes in response to faulty proline catabolism.

In this study, a relationship between proline and glucose catabolism, specifically the PPP, was revealed. Under normal conditions, HG is predominately fluxed into glycolysis and the Krebs cycle, which favors ROS production. Nevertheless, the failure of proline catabolism activates GSPD-1, which directs glucose flux into the PPP and produces NADPH for ROS elimination (Fig. S5). It is still unclear how HG-induced NADPH regulates ROS. In fact, it has been demonstrated that NADPH affects ROS levels *via* multiple mechanisms, such as the production of the reduced form of thioredoxin and the activation of catalase (33). In addition, P5C can be transported from mitochondria to cytosol, where it can be reconverted to proline with NADPH as the reducing power (Fig. S5). This may alleviate the P5C stress of *alh-6* mutants and thereby reduce ROS levels.

It is yet unknown how defective proline catabolism activates the PPP. The reconversion of P5C to proline depletes NADPH and produces NADP. Thus, the PPP may act as a feedback mechanism to restore NADPH levels in the context of faulty proline catabolism. In addition, since numerous other enzymatic reactions can alter the NADPH/NADP ratio, this raises the possibility that HG may become beneficial under such conditions, a notion that merits more investigation.

The reprogramming of glucose catabolism to the PPP pathway assists the animals in mitigating the deleterious ROS overproduction caused by the mutation of *alh-6/p5cdh*. The hyperprolinemia type II patients with *p5cdh* mutation may therefore benefit from the HG diet. Moreover, P5C buildup can induce cancer cell death. Further investigation into whether a similar cross talk between proline and glucose catabolism exists in cancer may yield new dietary intervention strategies for cancer therapy. We propose that dietary glucose levels may play a crucial role in determining the therapeutic efficacy for human disorders associated with faulty or altered proline catabolism.

## Experimental procedures

### *C. elegans* strains and maintenance

*C. elegans* were cultured on standard nematode-growth medium (NGM) seeded with indicated bacteria (34). The following strains were used in this study: the WT N2 Bristol, SPC321[*alh-6(lax105)*], CF1553[*sod-3p::gfp*], TJ356[*daf-16p::daf-16::gfp*], SJ4103[*myo-3p::gfp(mito)*], CF1038[*daf-16(mu86)*], and PS3551[*hsf-1(sy441)*]. The *gspd-1p::gspd-1::gfp* strain was generated by cloning the 2.4 kb promoter region and the full length of *gspd-1* genomic DNA into pPD95.79 plasmid, which was then injected into gonad by standard techniques. Double mutants were created by standard genetic techniques.

### RNAi treatment

Plasmids containing *gspd-1* dsRNA were transformed into RNAi-compatible OP50(xu363) bacteria (19). After seeding, RNAi was induced by IPTG at room temperature for 24 h. Then, L1 worms were introduced to RNAi plates to knock down *gspd-1* gene.

### Life span analysis

Life span assays were conducted as previously described (35). Briefly, synchronized L1 worms were introduced to NGM plates seeded with the indicated *E. coli* strains. Worms were transferred every day during the reproductive period. Worms that died of vulva burst, bagging, or crawling off the plates were censored.

### Quantitattive RT–PCR

Quantitative RT–PCR was conducted as previously described (36). Briefly, adult worms were collected, washed in M9 buffer, and then homogenized in Trizol reagent. RNAs were extracted according to the manufacturer’s instructions. RNA was reverse-transcribed to complementary DNA by using the RevertAid First Strand Complemenatry DNA Synthesis Kit (Thermo Fisher Scientific) after DNA contamination was digested with DNase I. Quantitative PCR was performed using SYBR Green, and data were collected using CFX Maestro Software (Bio-Rad). The expression of *snb-1* was used for sample normalization. Primer sequences are listed in Table S2.

### Fluorescent microscopy

Fluorescent analysis was performed as previously described (37).To analyze GFP fluorescence, adult worms were paralyzed with 1 mM levamisole, and fluorescent microscopic images were captured using Nikon NIS-Elements software or Leica LAS X software after being mounted on slides. To analyze mitochondrial morphology, *myo-3p*::GFP(mito) worms were mounted on slides, and the shapes of GFP-positive mitochondria were scored as tubular, intermediate, or fragmented. To study mitochondrial membrane potential, worms were moved to NGM plates with 100 nM MitoTracker Red CMXRos and incubated in the dark for 12 to 16 h before imaging. To study the DAF-16 nuclear occupancy, the nuclear localization levels of GFP were scored. Briefly, no nuclear GFP, GFP signal in the nucleus of anterior or posterior intestine cells, and nuclear GFP in all intestinal cells are categorized as low, medium, and high expression, respectively. To study *sod-3p*::GFP, fluorescence intensities were analyzed using ImageJ 1.53e software (NIH).

### Biochemical quantifications

To measure glucose levels, about 1000 age-synchronized day 2 adult worms were collected and washed three times with M9 buffer. Then, they were homogenized, and the supernatant was recovered after centrifugation. The glucose levels were determined using Tissue Glucose Content Assay Kit (Applygen) and normalized to the total protein contents.

ROS levels were determined using the H2DCFDA method. Worms were collected and washed three times with M9 buffer. Then, the H2DCFDA reaction solution was then added to the worms at a final concentration of 25 μM and incubated at room temperature for 8 h in the dark. Fluorescent intensity was measured using a BioTek multifunctional microplate reader.

To quantify NADPH/NADP and GSH/GSSG, about 2000 age-synchronized day 2 adult worms were collected. The NADPH/NADP and GSH/GSSG ratios were determined using the NADP+/NADPH Assay Kit and the GSH/GSSG Assay Kit (Beyotime), respectively, and normalized to the total protein concentrations.

To measure the GSPD-1/G6PDH activity, about 1000 age-synchronized day 2 adult worms were collected. The GSPD-1/G6PDH activity was determined using the G6PDH Activity Assay Kit (Beyotime) and then normalized to the total protein concentrations.

### Statistics

Data are presented as mean ± SD. GraphPad Prism 8 software (GraphPad Software, Inc) was used for statistical testing. Survival data were analyzed using the log-rank (Mantel–Cox) test. The mitochondrial morphology and nuclear accumulation of DAF-16::GFP were analyzed using the Chi-square and Fisher’s exact test. Figures 1, *B*, *D* and *H*, 2*G*, 3, *A* and *E*, 4*D*, S1*A*, S2*E*, S3, *D*, and *J* were analyzed using two-way ANOVA. Figures 3*G* and S3*I* were analyzed using one-way ANOVA. Figures 2, *B*–*D*, 4, *E* and *F*, and S2*F* were analyzed using multiple *t* tests. Figures 2, *E* and *H* and S3, *B*, *E* and *H*, and S4*G* were analyzed using the Student's *t* test. *p* < 0.05 was considered significant.

## Data availability

All data are contained within the article.

## Supporting information

This article contains supporting information.

## Conflict of interest

The authors declare that they have no conflicts of interest with the contents of this article.
