# Supplementary material for: The impact of glucose on mitochondria and life span is determined by the integrity of proline catabolism in Caenorhabditis elegans
Source: J Biol Chem. 2023 Jan 7;299(2):102881. doi: 10.1016/j.jbc.2023.102881 (PMC9932108; doi:10.1016/j.jbc.2023.102881)
Supplement: Supporting information [file mmc1.pdf]

The impact of glucose on mitochondria and lifespan is determined by the integrity of proline catabolism in *Caenorhabditis elegans*

Xi Feng[1], Xinyu, Wang[1], Lei Zhou[1, 2], Shanshan Pang[1], and Haiqing Tang[1, *]

[1]School of Life Sciences, Chongqing University, Chongqing, 401331, China

[2]State Key Laboratory of Silkworm Genome Biology, Key Laboratory of Sericultural Biology and Genetic Breeding, Ministry of Agriculture and Rural Affairs, College of Sericulture, Textile and Biomass Sciences, Southwest University, Chongqing, 400715, China

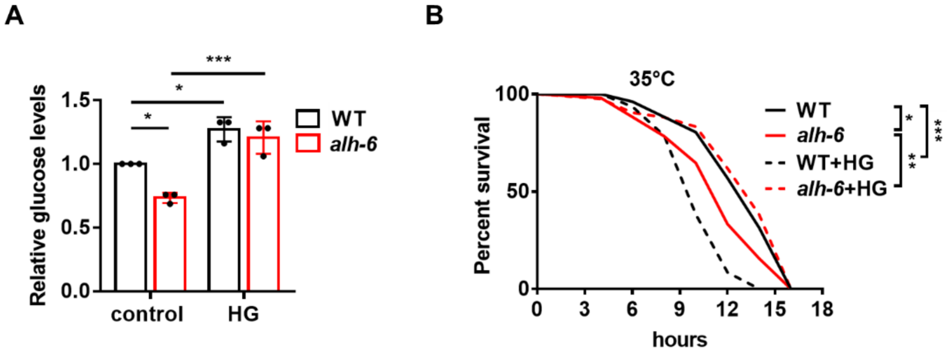

Figure S1. Dietary glucose normalizes the stress resistance of *alh-6* mutants. (A) Glucose levels in WT and *alh-6* mutants fed the HG diet. Data are from 3 independent experiments and normalized to the control values within the same experiment. (B) The effects of the HG diet on the stress resistance of WT and *alh-6* mutants fed OP50 diet. Data are represented as mean ± SD. $*p < 0.05$, $**p < 0.01$, $***p < 0.001$.

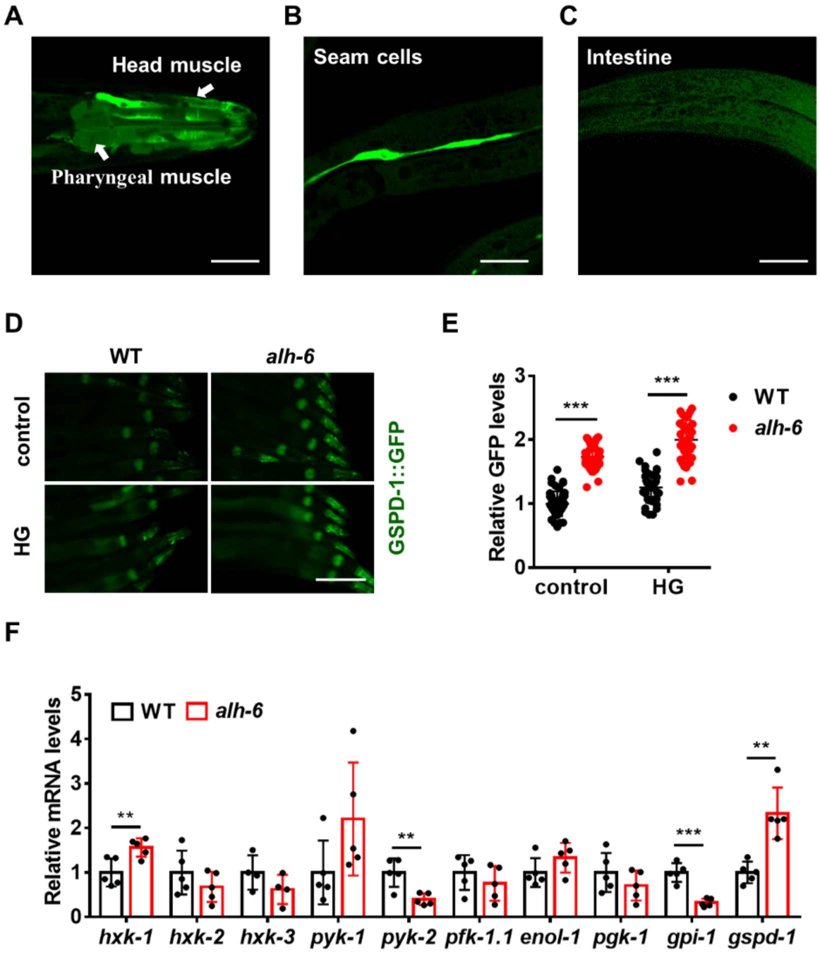

Figure S2. Regulation of glucose metabolic genes in *alh-6* mutants fed the HG and HT115 diets. (A-C) The GSPD-1::GFP signals are abundant in the pharyngeal muscle (A), head muscle (A), and seam cells (B), and substantially less abundant in the intestine (C). Scale bar = 25 μm. (D-E) Effects of the HG diet on the expression of GSPD-1::GFP in WT and *alh-6* mutants. Panel (D): representative images. Scale bar = 100 μm. Panel (E): quantification data. n = 37 worms per condition. (F) The expression of glucose metabolic genes in *alh-6* mutants fed HT115 bacteria. n = 5 biologically independent samples per condition. Data are represented as mean ± SD. $*p < 0.05$, $**p < 0.01$, $***p < 0.001$.

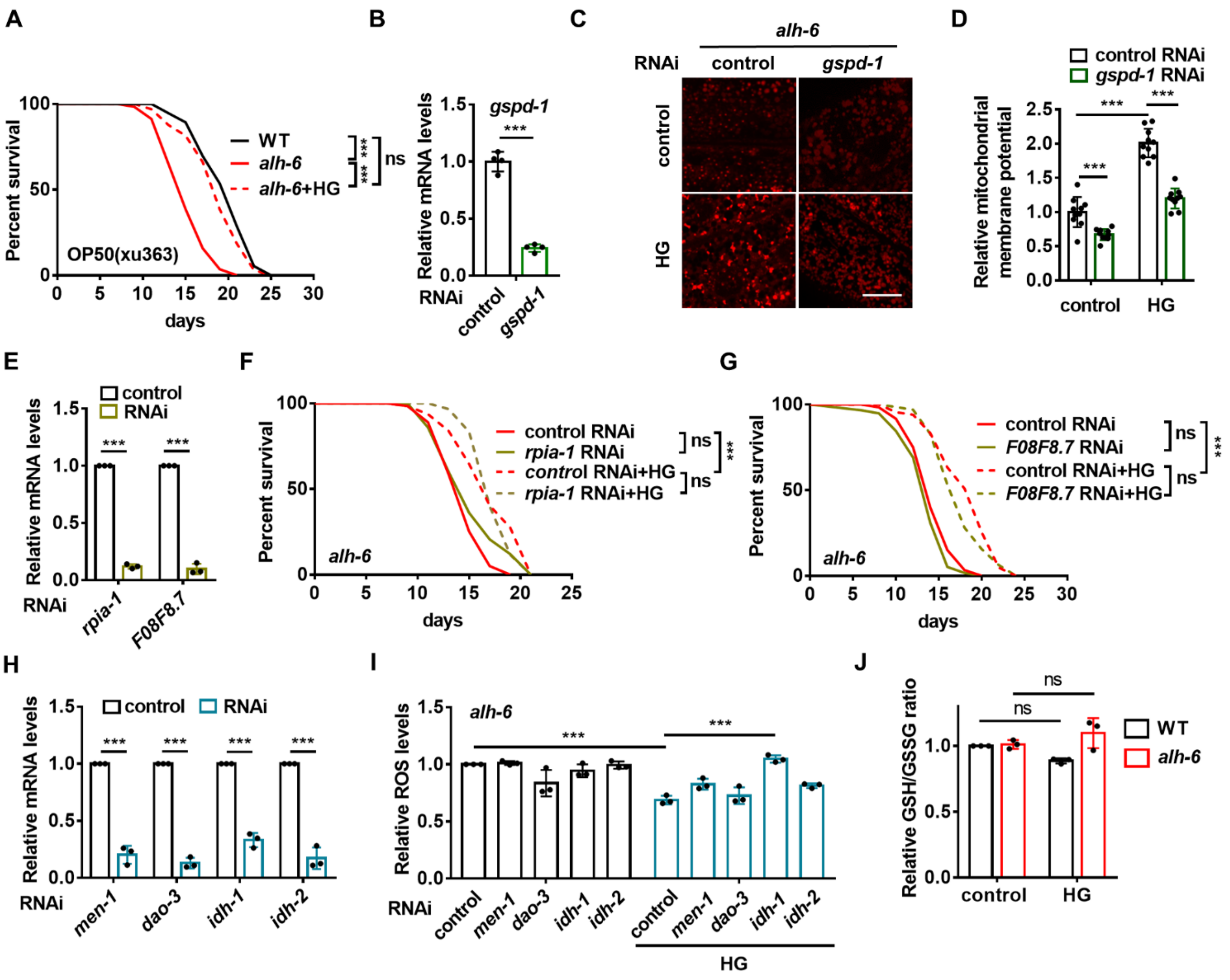

Figure S3. The protective effects of the HG diet in *alh-6* mutants are specifically mediated by GSPD-1. (A) The lifespan of *alh-6* mutants fed OP50(xu363) with or without HG. (B) Knockdown efficacy of *gspd-1* RNAi via OP50(xu363). n = 4 biologically independent samples per condition. (C-D) *gspd-1* RNAi suppresses the protective effects of the HG diet on mitochondrial membrane potential in *alh-6* mutants fed OP50(xu363) bacteria. Panel (C) shows the representative images (Scale bar = 12 µm) and panel (D) shows the semi-quantification data. n = 10 worms per condition. (E) Knockdown efficacy of the *C. elegans* non-oxidative arm genes *rpia-1/RPIA and F08F8.7/RPE.* Data are from 3 independent experiments and normalized to the control values within the same experiment. (F-G) RNAi of *rpia-1* (F) and *F08F8.7* (G) has no effects on the lifespan of *alh-6* mutants fed the HG diet. (H) Knockdown efficacy of the NAPDH-producing genes *men-1/ME1, dao-3/MTHFD, idh-1/IDH1*, and *idh-2/IDH2.* Data are from 3 independent experiments and normalized to the control values within the same experiment. (I) The effects of the NAPDH-producing genes on the ROS levels of *alh-6* mutants fed the HG diet. Data are from 3 independent experiments and

normalized to the control values within the same experiment. (J) The HG diet has no effects on the GSH/GSSG ratio in WT and *alh-6* mutants. Data are from 3 independent experiments and normalized to the control values within the same experiment. Data are represented as mean ± SD. ∗∗∗p < 0.001.

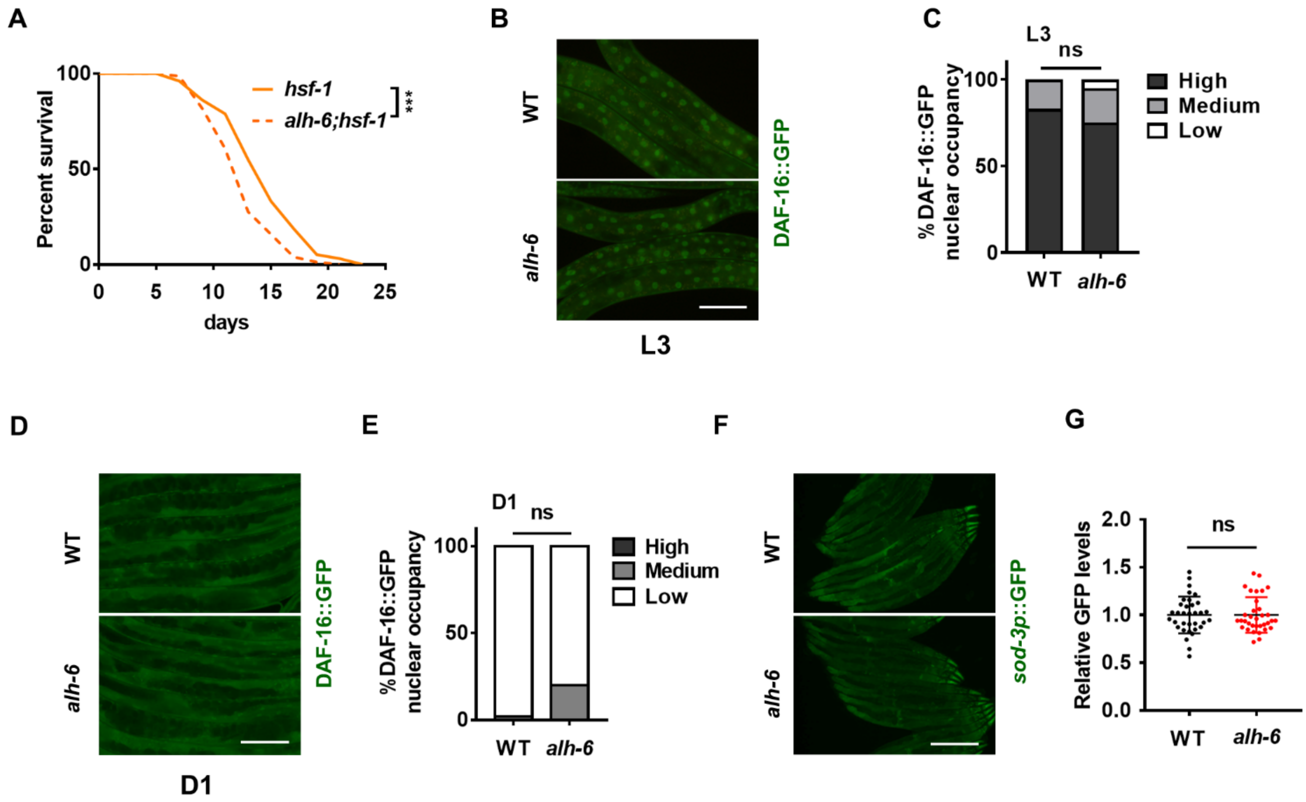

Figure S4. Effects of the *alh-6* mutation on DAF-16 activity. (A) The *alh-6* mutation shortened the lifespan of *hsf-1* mutants. (B-E) The effects of the *alh-6* mutation on DAF-16::GFP nuclear occupancy in worms at L3 stage (with spontaneous DAF-16 nuclear accumulation) (B-C) and day 1 adult stage (without DAF-16 nuclear accumulation). Panels (B, D): representative images. Scale bar = 50 μm. Panels (C, E): semi-quantification data. n = 30 worms per condition. (F-G) The effect of the *alh-6* mutation on *sod-3p*::GFP expression. Panel (F): representative images. Scale bar = 200 μm. Panel (G): semi-quantification data. n = 34 worms per condition. Data are represented as mean ± SD. ∗∗∗p < 0.001.

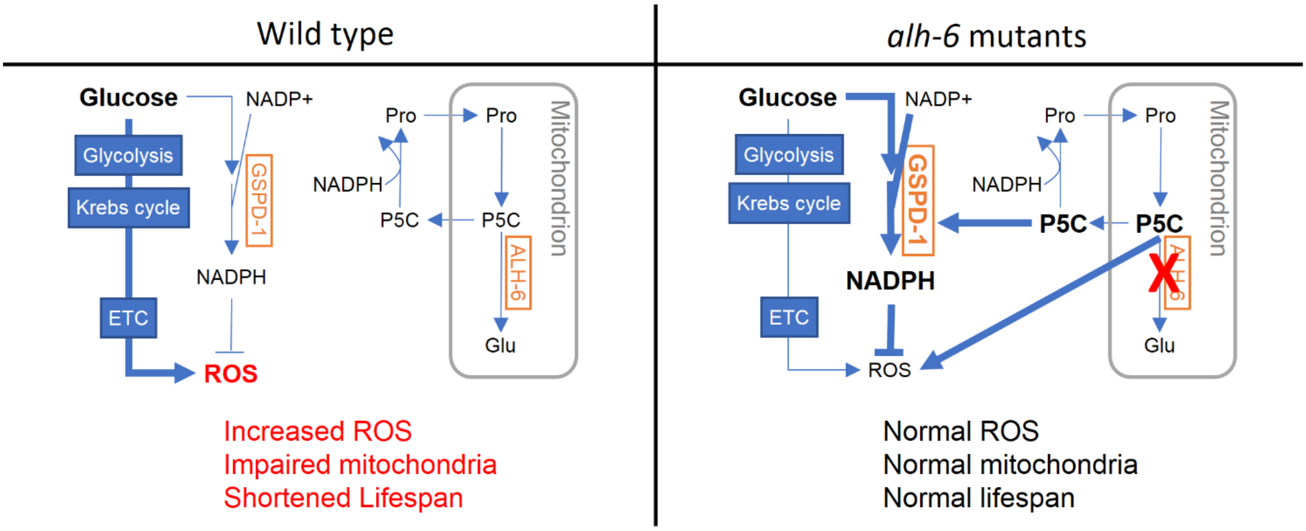

Figure S5. The communication between proline and glucose catabolism controls ROS homeostasis and influences mitochondrial function and lifespan. Normal glucose flow into glycolysis and the Krebs cycle in WT animals generates ATP via the electron transport chain (ETC), which produces ROS as a byproduct, hence damaging mitochondria and shortening lifespan. When *alh-6/p5cdh* is mutated, the mitochondrial buildup of metabolic intermediate P5C activates GSPD-1/G6PDH, the rate-limiting enzyme of the PPP. This action directs glucose flux towards the PPP, producing NADPH to combat ROS and protect mitochondria.

Table S1. Survival data.

Figures	Strain/Treatment	Mean survival time ± SEM (days)	# Worms Censored/Total	P value
**1C**	WT	17.69 ± 0.47	4/74	
	alh-6	14.06 ± 0.62	11/70	<0.001[a]
	WT + HG	13.87 ± 0.46	15/69	<0.001[a]
	alh-6 + HG	16.60 ± 0.40	17/71	0.0286[b]
**3D**	*alh-6* control RNAi	15.24 ± 0.33	4/78	
	alh-6 gspd-1 RNAi	11.37 ± 0.27	6/72	<0.001[b]
	alh-6 control RNAi + HG	18.64 ± 0.50	7/82	<0.001[b]
	alh-6 gspd-1 RNAi + HG	14.25 ± 0.30	3/77	<0.001[c]
**3F**	WT control RNAi	19.79 ± 0.41	0/71	
	WT *gspd-1* RNAi	15.02 ± 0.43	5/77	<0.001[a]
	alh-6 control RNAi	17.34 ± 0.49	3/82	0.0023[a]
	alh-6 gspd-1 RNAi	11.92 ± 0.34	2/60	<0.001[b]
**4A**	WT	18.48 ± 0.38	6/85	
	alh-6	14.50 ± 0.42	10/72	<0.001[a]
	daf-16	13.85 ± 0.29	3/93	<0.001[a]
	alh-6;daf-16	18.90 ± 0.47	11/73	<0.001[b]
**S1B**	WT	13.06 ± 0.39 (hours)	1/52	
	alh-6	11.57 ± 0.44 (hours)	0/51	0.0148 [a]
	WT + HG	10.49 ± 0.37 (hours)	1/38	<0.001[a]
	alh-6 + HG	13.38 ± 0.47 (hours)	0/45	0.0013[b]
**S3A**	WT	19.82 ± 0.41	7/63	
	alh-6	15.18 ± 0.33	5/62	<0.001[a]
	alh-6 + HG	18.85 ± 0.42	6/71	0.1236 [a] <0.001[b]
**S3F**	*alh-6* control RNAi	14.53 ± 0.28	7/67	
	alh-6 rpia-1 RNAi	15.3 ± 0.4	7/66	0.0552[b]
	alh-6 control RNAi + HG	17.17 ± 0.4	5/67	<0.001[b]
	alh-6 rpia-1 RNAi + HG	17.63 ± 0.25	4/64	0.7274[c]
**S3G**	*alh-6* control RNAi	14.47 ± 0.32	2/62	

	alh-6 F08F8.7 RNAi	13.68 ± 0.35	4/62	0.1299[b]
	alh-6 control RNAi + HG	18.45 ± 0.42	6/69	<0.001[b]
	alh-6 F08F8.7 RNAi + HG	17.63 ± 0.38	5/69	0.1148[c]
**S4A**	*hsf-1*	14.64 ± 0.38	13/111	
	alh-6; hsf-1	12.69 ± 0.31	6/81	<0.001[d]

a vs WT

b vs *alh-6*

c vs *alh-6* control RNAi + HG

d vs *hsf-1*

Table S2. qPCR primers.

**Primers**	**Sequences**
gspd-1 Forward	CCAATACGACACTTCTGAA
gspd-1 Reverse	AAGAGCGAGGTAGTAGAG
hxk-1 Forward	GCTGGAATCGCTATGTTG
hxk-1 Reverse	TGTTGGATAAGTTGGATGGA
hxk-2 Forward	CAACGCTACGGAGGATTA
hxk-2 Reverse	AGAATTAACGCCAGGATGA
hxk-3 Forward	GCCTCCAACGATTCCTAC
hxk-3 Reverse	TGCCGAACGAATTGACAA
pfk-1.1 Forward	TGCTGCTCAACTGAAGAA
pfk-1.1 Reverse	AGAATTATTGTGCTGTGATACG
aldo-2 Forward	GAATCCACTGGCACAATC
aldo-2 Reverse	TGACGATACTTCTGACGAT
enol-1 Forward	AGTCTACCACCACTTGAA

enol-1 Reverse	TCCTTCCTTGTTGTCTTG
pgk-1 Forward	AAGAAGTATAACACGGAAGATAAG
pgk-1 Reverse	ATCAACTCCTGGCAGAAC
gpi-1 Forward	TGGAATGAGCCCTGAATC
gpi-1 Reverse	ATGGTGTGACAACTGGAA
pyk-1 Forward	AGGAACTGCTGTCAATCTT
pyk-1 Reverse	ATAAGTCCGTCATCAATGTAGA
pyk-2 Forward	CGTGGATTCATTCATCTT
Pyk-2 Reverse	GGCAACTACAATTCTCAT
mtl-1 Forward	ATGGCTTGCAAGTGTGACTG
mtl-1 Reverse	CACATTTGTCTCCGCACTTG
sod-3 Forward	CCAACCAGCGCTGAAATTCAATGG
sod-3 Reverse	GGAACCGAAGTCGCGCTTAATAGT
hsp-16.1 Forward	GCAGAGGCTCTCCATCTGAA
hsp-16.1 Reverse	GCTTGAACTGCGAGACATTG
ges-1 Forward	AGCAACAAGGAAGGGTCGTA
ges-1 Reverse	CCGATGATCTCCGAAATGAA
C32H11.4 Forward	TTACTTCCCATCGCCAAAGT
C32H11.4 Reverse	CAATTCCGGCGATGTATGAT
dod-24 Forward	TGTCCAACACAACCTGCATT
dod-24 Reverse	TGTGTCCCGAGTAACAACCA
dod-17 Forward	GTTGGATTGTGACAGTTC

dod-17 Reverse	TTGAGTAGTCCGAATAAGAT
rpia -1 Forward	TCATCTGTGTTCCAACCA
rpia -1 Reverse	TGTCCGTCAACTTCATCA
F08F8.7 Forward	TTGACGGAGGAGTAACAC
F08F8.7 Reverse	ATGGCGACAGATTGATCT
men-1 Forward	AATGCGTATCGGTTGTTG
men-1 Reverse	AGAGCTTCTTCTTGGTGAT
dao-3 Forward	TTGTTCAGGTTGGAAATCG
dao-3 Reverse	CCTTGAGTGATGGTGTCT
idh-1 Forward	GATTGATGACATCTGTGCTT
idh-1 Reverse	ATTGGATTCGTGGAGGTT
idh-2 Forward	TTGAATACAGAGACGAGACA
idh-2 Reverse	TGAGAGCCACATCTTCTTAA
